# Case report: atypical presentation of vancomycin induced DRESS syndrome: a case report and review of the literature

**DOI:** 10.1186/s12890-017-0564-6

**Published:** 2017-12-28

**Authors:** Olivia Wilcox, Mohamed Hassanein, John Armstrong, Nader Kassis

**Affiliations:** 1 Department of Internal Medicine, Michigan State University, East Lansing, MI USA; 2 Pulmonary Critical Care, Sparrow Medical Group, Lansing, MI USA; 3 Nephrology, Sparrow Medical Group, Lansing, MI USA

**Keywords:** Drug reaction, Eosinophilia, Dress, Vancomycin, Acute respiratory distress syndrome, ARDS

## Abstract

**Background:**

Drug reaction with eosinophilia and systemic symptoms (DRESS) is a severe hypersensitivity drug reaction involving the skin and multiple internal organ systems. The symptoms typically present with fever and skin rash, and rapidly progress to multiple organ failures. Vancomycin is a rare drug to cause DRESS syndrome with 23 cases reported to date.

**Case presentation:**

We described a case of a 39 year-old man who was treated with vancomycin for osteomyelitis of the foot. The patient subsequently developed acute respiratory distress syndrome (ARDS) followed by rash and acute interstitial nephritis. These symptoms were improved by withdrawal of vancomycin and a pulsed corticosteroid regimen. According to the European Registry of Severe Cutaneous Adverse Reaction Criteria (RegiSCAR) (Kardaun et al, British Journal of Dermatology, 169:1071-1080, 2013), the probability of vancomycin induced DRESS syndrome was scored as “Definite”. A literature search of vancomycin induced DRESS syndrome was also performed and the overall pulmonary involvement was estimated as 5%. To our knowledge, this was the first case reported with pulmonary involvement as the initial symptom.

**Conclusion:**

This is the first case to report pulmonary manifestation as the initial symptom in vancomycin induced DRESS syndrome. Prompt recognition of this entity can expedite proper treatment and hasten recovery.

**Electronic supplementary material:**

The online version of this article (10.1186/s12890-017-0564-6) contains supplementary material, which is available to authorized users.

## Background

Drug reaction with eosinophilia and systemic symptoms (DRESS) is a severe hypersensitivity drug reaction involving the skin and multiple organs. It was first described as “Dilantin syndrome” [1], and later as “Drug-induced pseudolymphoma and hypersensitivity syndrome” [2]. The term DRESS Syndrome was first proposed by Bocquet H et al. in 1996 [3]. Symptoms typically present with a skin rash [4], and are accompanied by fever, eosinophilia, atypical lymphocytosis, and multiple organ failures including the liver, kidneys and lungs [5]. The hallmark of DRESS is the prolonged latency, with symptoms appearing after 2 to 6 weeks from the initial drug exposure [6]. The pathogenesis is not fully understood, but is thought to be related to immunosuppression upon drug hypersensitivity reaction, and an underlying viral infection, such as human herpesvirus 6 (HHV-6). This immunosuppression may in turn lead to more severe systemic drug reaction [5, 7, 8].

Drugs associated with DRESS syndrome include, but are not limited to, anticonvulsants, antimicrobials, antivirals, and antidepressants [6]. Vancomycin is an uncommon drug to cause DRESS syndrome, with 23 cases reported to date (Table 1). Described here is a case of vancomycin induced DRESS syndrome with an atypical presentation, which manifested initially with acute respiratory distress syndrome (ARDS), followed by rash, fever, and acute interstitial nephritis. To our knowledge, this is the first case reported with pulmonary involvement as the initial symptom.

**Table 1:** Literature review of vancomycin induced DRESS syndrome

Year	Author, Country	Age/Sex	Preceding events	Initial Symptoms	Organ system involved	Treatment and Outcome
1997	Marik PE, US [22]	51/M	Sepsis	Rash	Kidneys	Corticosteroids, survival
2005	Yazganoǧlu K, Turkey [23]	56/W	Methicillin-resistant *Staphylococcus aureus* (MRSA) bacteremia	Fever, Rash	Liver, kidneys	Corticosteroids, survival
2005	Zuliani E, Switzerland [24]	41/W	Bacterial endocarditis	Rash, Fever	Kidneys, liver	Corticosteroids and cyclosporine, survival
2006	Kwon H-S, Korea [25]	50/M	Vertebral osteomyelitis	Rash, fever	Kidneys	Corticosteroids, survival
2007	Tamagawa-Mineoka R, Japan [26]	52/W	MRSA, ear surgery	Rash, fever	Liver, kidneys	Pulsed Corticosteroids, survival
2008	Vauthey L, Switzerland [27]	60/W	MRSA, leg amputation	Rash, fever, periorbital edema	Liver	Corticosteroids, survival
2009	Mennicke M, Switzerland [28]	60/M	*Staphylococcus aureus* bacteremia	Fever, rash	Liver	Patient deceased after liver transplantation
2010	Vinson AE, US [29]	14/M	Spinal surgery infection	Fever, Rash, vomiting	Liver	Corticosteroids, survival
2011	O’Meara P, Canada [30]	60/M	MRSA, pelvic surgery	Rash	Liver, kidneys, Lungs	Corticosteroids, survival
2012	Blumenthal KG, US [31]	65/M	Hemolytic Group B *Streptococcus* empyema, Esophageal perforation	Rash	Liver, kidneys, Lungs	Corticosteroids, survival
2012	Blumenthal KG, US [31]	40/M	*Propionibacterium* and *Peptostreptococcus*, prosthetic shoulder	Fever, Rash	Liver, kidneys	Corticosteroids, survival
2012	Blumenthal KG, US [31]	48/W	Coagulase-negative *Staphylococcus*, shoulder surgery	Rash	Liver, gastrointestinal track	Treatment and outcome not clearly mentioned.
2012	Blumenthal KG, US [31]	74/M	Hand trauma	Rash	Liver	Treatment and outcome not clearly mentioned.
2012	Blumenthal KG, US [31]	51/M	Osteomyelitis of the finger	Rash, fever	Liver	Corticosteroids, survival
2012	Díaz-Mancebo R, Spain [32]	74/W	*Staphylococcus epidermidis*, spondylodiscitis	Rash, Renal injury	Kidneys, liver, lungs	Pulsed corticosteroids, survival
2014	Young S, Australia [33]	24/M	*Corynebacterium jeikeium*, septic arthritis	Rash	Liver	Corticosteroids, survival
2014	Young S, Australia [33]	48/W	Osteomyelitis	Rash, hepatic injury	Liver	Corticosteroids, survival
2014	Young S, Australia [33]	59/M	MRSA, wound infection	Rash	Liver	Corticosteroids, survival
2015	Güner MD, Turkey [34]	73/M	Infected left hip prosthesis	Fever	Kidneys	Corticosteroids, survival
2015	Güner MD, Turkey [34]	72/W	Infected left knee prosthesis	Fever	Kidneys, liver	Corticosteroids, survival
2016	Kim KM, Japan [35]	11/M	Parotitis	Rash, proteinuria	Kidneys	Corticosteroids, survival
2016	Miyazu D, Japan [36]	79/M	MRSA, femur fracture	Rash, fever	Lung	Corticosteroids, survival
2016	Webb PS, UK [37]	73/M	Septic shock	Fever, rash	Kidneys, heart	Corticosteroids, survival

## Case presentation

**Fig. 1:**
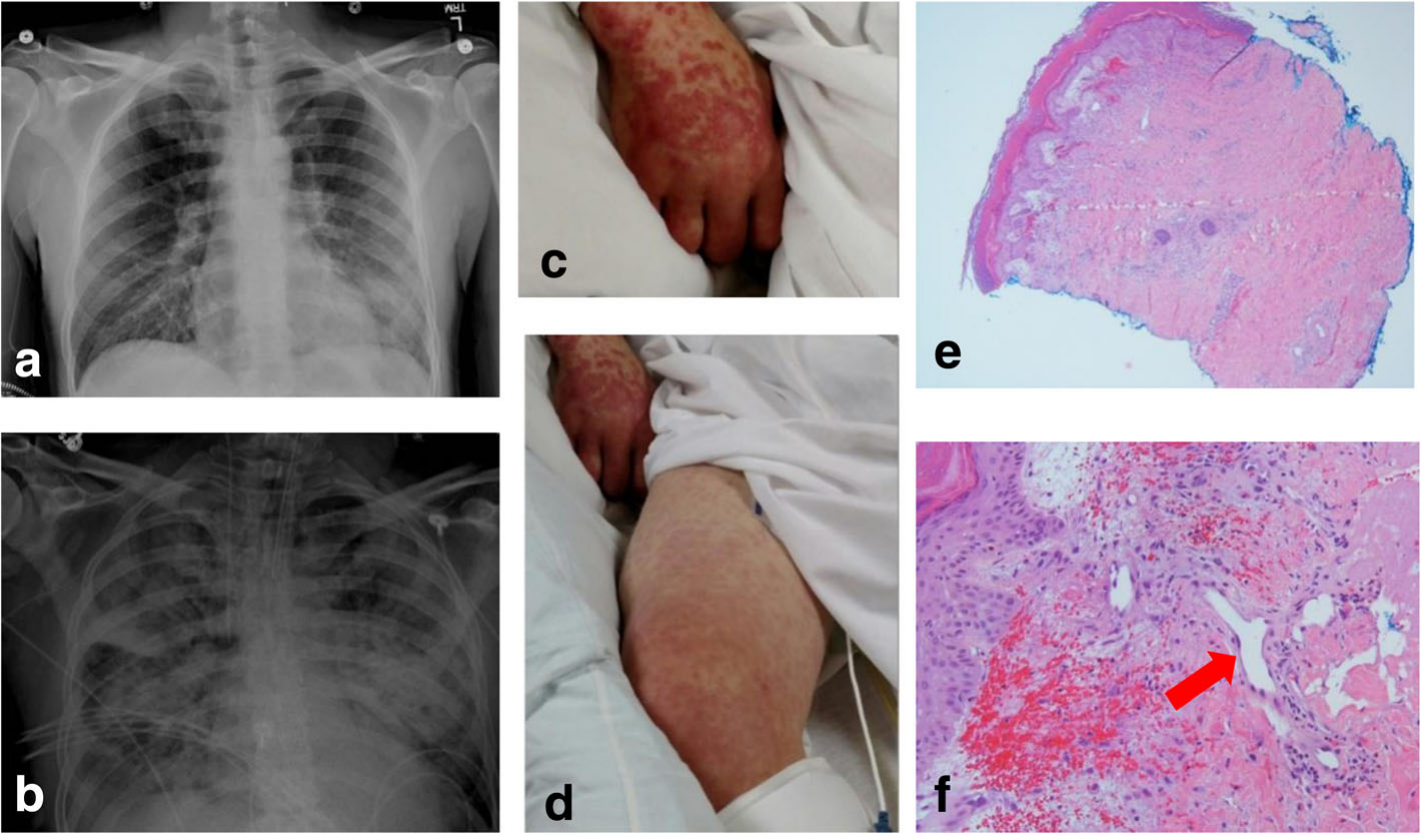
Clinical and imaging manifestation of the case. **a** Chest X-Ray on day 1 showing mild left lower lobe airspace opacity; **b** Chest X-Ray on day 3 showing diffuse pulmonary infiltrate; **c**&**d** Diffuse rash on day 8; **e** 3 mm punch biopsy of the dorsum of the right hand; **f** Absence of fibrin and microthrombi deposition in the vessel lumen (arrow)

A 39 year-old man presented to our institution with fever, chills, and shortness of breath for 5 days. He had a past medical history of type I diabetes, seizures, and hyperlipidemia. His home medications included atorvastatin, insulin, lamotrigine and pregabalin. He had allergy to cephalexin, with itchiness as the only allergic symptom. He also had a right calcaneus fracture with open reduction internal fixation, and he was recently diagnosed with osteomyelitis of the right calcaneus. He was started with parenteral vancomycin 3 weeks prior to admission. On physical exam at admission, he was noted to be febrile (102.6 °F), with severe shortness of breath. There was no evidence of rash, renal injury, lymphadenopathy or hepatosplenomegaly. Chest x-ray in the emergency department (ED) revealed a mild left lower lobe airspace opacity (Fig. 1a). He was placed on vancomycin and levofloxacin, and was admitted for possible community acquired pneumonia. At admission, his laboratory tests, however, were not suggestive of infection, with Leukocyte count 6700/mm^3^ (Neutrophils: 72.1%, Lymphocytes: 15.4%, Monocytes: 7.9%, Eosinophils: 4%, Basophils: 0.6%), Absolute eosinophil count 270/uL (within normal range), Lactate 1.0, and with only mildly elevated C-reactive protein 3.4 (CRP) (0.0–1.0 mg/dL), and mildly elevated Sedimentation rate (SED) 20 (0–15 mm/h). Orthopedics was also consulted in the ED and determined that his osteomyelitis was controlled and localized, with no acute intervention needed.

**Fig. 2:**
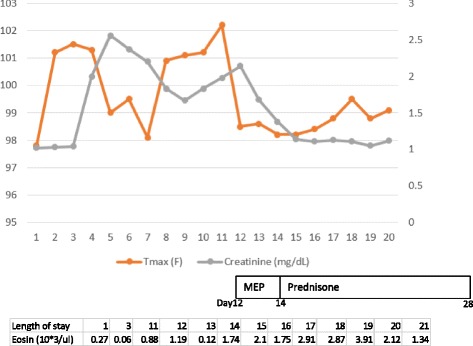
Laboratory data and response to corticosteroid treatment. Tmax – temperature maximum; Eosin - eosinophils; MEP – Methylprednisolone

Hospital day 2–3: He developed ARDS with pre-intubation PaO2/FiO2 ratio 82 (100% FiO2) and post-intubation PaO2/FiO2 ratio 109 (70% FiO2 and PEEP of 10 cm H20) (Fig. 1b). He was placed on vancomycin, piperacillin - tazobactam and levofloxacin for possible sepsis. 10–20 ml bronchoalveolar lavage (BAL) fluid was obtained via bronchoscopy and revealed serosanguineous fluid with 10–15 white blood cell per high power field and with no organisms identified. Polymerase chain reaction test was obtained via nasopharyngeal swab and was negative for Influenza and Respiratory Syncytial Virus. Blood culture was negative for organisms. There was a questionable convulsion episode witnessed by the family and he was started on levetiracetam due to a history of seizure. An echocardiogram was performed and indicated normal cardiac function (ejection fraction 60%) and normal right ventricular diastolic pressure (36.8 mmHg). Day 4–5: he developed new onset acute renal injury (AKI) and new morbilliform rash in bilateral hands and knees; vancomycin, piperacillin - tazobactam and levofloxacin were discontinued, and meropenem and daptomycin were started. Day 6–11: the rash progressed to the entire body (sparing face) and became indurated with associated edema (Fig. 1c, d); new onset eosinophilia occurred with a peak of 4000/uL; fever and leukocytosis persisted despite treatment with antibiotics. AKI workup indicated interstitial nephritis primarily based on positive urine eosinophil (7%), and negative autoimmune workup including Myeloperoxidase Antibody (<0.2), Proteinase 3 Antibody (<0.2), Antinuclear Antibody (negative), C3 complement 115 (77–166), and C4 complement 34 (18–52); Urine protein to creatinine ratio was 0.66 (normal). Lumbar puncture and magnetic resonance imaging of the brain excluded bacterial meningitis, Cryptococcus, Varicella Zoster Virus, Herpes Simplex I/II and Toxoplasmosis. Computed tomography of the abdomen and pelvis revealed no intra-abdominal infectious source. A skin biopsy showed perivascular lymphocytes, occasional neutrophils, scattered eosinophils, and the absence of intraluminal microthrombi (Fig. 1e, f). Day 12–14: patient continued to be in critical condition with persistent fever, leukocytosis and negative infection workup. The decision was made to treat as DRESS syndrome with a pulsed corticosteroid regimen, methylprednisolone 125 mg (approximately 1.25 mg/kg daily, body weight 99.3 kg) for 3 days. The patient responded well. He had resolution of the fever, and clinical improvement of ventilation. His renal function recovered in 2 days, and the diffuse rash was reduced to trace erythema in 5 days. The response to the corticosteroids is illustrated Fig. 2 in further details. He was treated with tapering oral prednisone for additional 2 weeks, and was discharged to inpatient rehabilitation facility, and then home. A three-month phone follow up revealed independent daily living skills and improved exercise tolerance. A time table of the hospital course can be found in the Additional file 1.

## Discussion and conclusions

DRESS syndrome is a dermatological emergency with mortality approximately 10% [9]. It has an estimated incidence ranging between 1 in 1000 and 1 in 10,000 drug exposures [3, 10]. The pathogenesis of DRESS syndrome is not well understood, but is hypothesized to be an immunological reaction with possible viral involvement [11]. It is associated with decreasing circulating B cells and serum immunoglobulin level at the early stage of the disease [11]. This immunosuppression may lead to viral reactivations, such as from HHV-6, which may in turn lead to more severe systemic immune reaction. DRESS is also associated elevation of inflammatory cytokines during the course of the disease, in particular, Interleukin 5 has been reported to peak several days before the peak of eosinophilia [12]. We speculate that these inflammatory cytokines may have contributed to the organ injury, and subsequently promoted eosinophilia.

Due to the variability of clinical presentation of DRESS, a scoring system, the European Registry of Severe Cutaneous Adverse Reaction Criteria (RegiSCAR), was proposed to aid diagnosis [13]. The patient scored seven points according to the RegiSCAR criteria, including fever (one point), eosinophilia greater than 1500/uL (two points), skin involvement and biopsy suggesting drug eruption (two points), renal and lung involvements (two points), and negative testing for ANA, blood culture and hepatitis. This score categorized the probability of vancomycin induced DRESS syndrome as “Definite”.

We also considered other diagnoses involving hypereosinophilia and multiple organ failures. Eosinophilic Granulomatosis with Polyangiitis (EGPA), previously Churg-Strauss, is a necrotizing vasculitis associated with severe asthma and eosinophilia [14]. Features of EGPA include elevated Antineutrophil Cytoplasm Antibody (ANCA) (30–70% cases), tissue eosinophilia in BAL fluid, and intraluminal microthrombi and fibrinoid necrosis in skin biopsy [14, 15]. The patient had negative ANCA, absence of eosinophils in the BAL fluid, and absence of intraluminal microthrombi in skin biopsy, making EGPA a less likely option. Hypereosinophilic syndrome (HES) is another entity which overlaps with DRESS. Secondary HES has a broad range of causes including parasitic infection, allergy and drug reactions. DRESS is currently classified as a subtype of drug induced HES [16]. Alternatively, primary HES is a myeloproliferative disorder and involves neoplastic eosinophils with cytogenetic abnormalities [16]. The peripheral blood smear of the patient did not demonstrate any lymphoid or myeloid lineage dysplasia that may be suggestive of primary HES. Stevens Johnson Syndrome and Toxic Epidermal Necrolysis (SJS/TEN) is a spectrum of severe drug reaction and features epidermal necrosis with epidermis detachment of at least 10% of body surface [17]. The rash in this case did not involve any bullae, desquamative or erosive lesions, and its appearance was not suggestive of SJS/TEN. Kawasaki disease is another condition characterized with fever, rash and systemic organ involvement. Kawasaki disease has well defined criteria including polymorphic rash, conjuctival changes and lymphadenopathy, which are not present in this case [18].

Vancomycin is a glycopeptide antibiotic with a half-life of 6 to 10 days [19]. It has activity against many gram-positive bacteria, and the gram-negative bacteria in the genus of Flavobacterium [19]. In the past few decades, due to the increasing prevalence of Methicillin-resistant *Staphylococcus aureus* (MRSA), parenteral vancomycin has been established for treating a variety of infections including sepsis, pneumonia, cellulitis, endocarditis and meningitis [20, 21]. Significant hypersensitivity reactions from vancomycin have been reported, including Linear IgA Bullous Dermatosis, DRESS, SJS/TEN [20]. We performed a literature search and identified 23 cases of vancomycin induced DRESS syndrome (Table 1) [22–37]. In addition, there are two articles in French and one article in German, which are not included in the discussion here. It is rare to involve pulmonary system and Miyazu D et al. have estimated pulmonary involvement to be 5% [36]. There are four cases reported to involve the pulmonary system and our case is the first one that presented with acute respiratory failure as the initial symptom [30–32, 36].

The mainstay of treatment for DRESS syndrome is withdrawal of the offending medication and treatment with corticosteroid. Due to vancomycin’s prolonged half-life, severe refractory cases may benefit from dialysis. One session of hemodialysis may remove up to 50% of plasma concentration of vancomycin [19]. A stepwise algorithm was proposed to treat DRESS syndrome with parenteral corticosteroid until resolution of fever and rash followed by oral corticosteroid for 4 to 6 weeks [38]. Rapid taper of corticosteroid can result in reoccurrence of symptoms and prolonged hospital stay [33]. A pulsed corticosteroid treatment has been suggested as an alternative treatment regimen, possibly due to decreased side effect [32]. We achieved prompt recovery with pulsed methylprednisolone at 1.25 mg/kg daily for 3 days. Higher doses of corticosteroids were also reported, including methylprednisolone 250 mg daily [32], and 500 mg daily [26], for 3 days. Higher dose of corticosteroids may be associated with an increased risk of agitation and difficulty weaning ventilation in intubated patient. Numerous long-term sequelae of DRESS syndrome has been reported, including infections, thyroiditis, type I diabetes, and acute interstitial nephritis [39].

In conclusion, this is the first case to report pulmonary manifestation as the initial symptom in vancomycin induced DRESS syndrome. Prompt recognition of this entity can expedite proper treatment and hasten recovery.

## Additional file

Additional file 1: Time table – Flow diagram - Flow diagram of important events during the hospital stay. (DOCX 52 kb)

## Abbreviations

AKI: Acute renal injury
ANCA: Antineutrophil Cytoplasm Antibody
ARDS: Acute respiratory distress syndrome
BAL: Bronchoalveolar lavage
DRESS: Drug reaction with eosinophilia and systemic symptoms
EGPA: Eosinophilic Granulomatosis with Polyangiitis
HES: Hypereosinophilic syndrome
HHV-6: Human Herpesvirus 6
MRSA: Methicillin-resistant *Staphylococcus aureus*
RegiSCAR: European Registry of Severe Cutaneous Adverse Reaction Criteria
