# Supplementary material for: Case report: atypical presentation of vancomycin induced DRESS syndrome: a case report and review of the literature
Source: BMC Pulm Med. 2017 Dec 28;17:217. doi: 10.1186/s12890-017-0564-6 (PMC5745618; doi:10.1186/s12890-017-0564-6)
Supplement: Additional file 1: — Time table – Flow diagram - Flow diagram of important events during the hospital stay. (DOCX 52 kb) [file 12890_2017_564_MOESM1_ESM.docx]

PMH: type I diabetes, seizures (controlled), hyperlipidemia, recent diagnosis of osteomyelitis of the foot; on vancomycin for 3 weeks prior to admission.

Allergy: cephalexin (itchiness)

Rash progressed to the entire body; new onset of eosinophilia; unremarkable MRI of the brain; negative LP analysis; negative autoimmune panel; skin biopsy consistent with drug eruption.

New acute renal injury and new focal morbilliform rash; discontinued all previous antibiotics; started meropenem and daptomycin.

Developed ARDS and intubated; treated as sepsis with vancomycin, piperacillin - tazobactam and levofloxacin; unremarkable BAL, negative blood culture and normal echocardiogram.

Initial Treatment: pneumonia treated with vancomycin and levofloxacin; no intervention for osteomyelitis per orthopedics.

Diagnostic Evaluations: left lower lobe airspace opacity on chest x-ray; CTA study negative for pulmonary emboli.

Physical Examination: febrile (102.6 °F); no rash; no lymphadenopathy or hepatosplenomegaly.

Current Illness: fevers; shortness of breath for 5 days.

#4-5

hysical Examination

Initial Treatment (and referrals if indicated)

Ongoing Interventions and Follow-up

Final Follow-up

Resolution of this Episode of Care

Ongoing Interventions and Follow-up

Ongoing Interventions and Follow-up

Diagnostic Evaluations

Diagnoses

Initial Treatment (and referrals if indicated)

Ongoing Interventions and Follow-up

Final Follow-up

Resolution of this Episode of Care

#6-11

hysical Examination

Initial Treatment (and referrals if indicated)

Ongoing Interventions and Follow-up

Final Follow-up

Resolution of this Episode of Care

Ongoing Interventions and Follow-up

Ongoing Interventions and Follow-up

Diagnostic Evaluations

Diagnoses

Initial Treatment (and referrals if indicated)

Ongoing Interventions and Follow-up

Final Follow-up

Resolution of this Episode of Care

#2-3

hysical Examination

Initial Treatment (and referrals if indicated)

Ongoing Interventions and Follow-up

Final Follow-up

Resolution of this Episode of Care

Ongoing Interventions and Follow-up

Ongoing Interventions and Follow-up

Diagnostic Evaluations

Diagnoses

Initial Treatment (and referrals if indicated)

Ongoing Interventions and Follow-up

Final Follow-up

Resolution of this Episode of Care

#1-2

hysical Examination

Initial Treatment (and referrals if indicated)

Ongoing Interventions and Follow-up

Final Follow-up

Resolution of this Episode of Care

Ongoing Interventions and Follow-up

Ongoing Interventions and Follow-up

Diagnostic Evaluations

Diagnoses

Initial Treatment (and referrals if indicated)

Ongoing Interventions and Follow-up

Final Follow-up

Resolution of this Episode of Care

Diagnoses: pneumonia

Hospital

Day #1

#12-14

#15- 28

Prednisone for two weeks; discharged to inpatient rehab and then home.

A three-month phone follow up revealed improved exercise tolerance and independent daily living skills.

Methylprednisolone 125 mg daily (1.25mg/kg) for three days, resulting in marked clinical improvement.
